# Lung function and exercise capacity 6 months after hospital discharge for critical COVID-19

**DOI:** 10.1186/s12890-022-02023-w

**Published:** 2022-06-22

**Authors:** Salla Kattainen, Anna Lindahl, Tuula Vasankari, Henriikka Ollila, Kirsi Volmonen, Päivi Piirilä, Paula Kauppi, Juuso Paajanen, Hanna-Riikka Kreivi, Linda Ulenius, Tero Varpula, Miia Aro, Jere Reijula, Johanna Hästbacka

**Affiliations:** 1 Division of Intensive Care, Department of Anaesthesiology, Intensive Care and Pain Medicine, Helsinki University Hospital, Helsinki, Finland; 2 Faculty of Medicine, University of Helsinki, Helsinki, Finland; 3 Finnish Lung Health Association, Helsinki, Finland; 4 Department of Pulmonary Diseases and Clinical Allergology, Faculty of Medicine, University of Turku, Turku, Finland; 5 Radiology, HUS Diagnostic Center, University of Helsinki and Helsinki University Hospital, Helsinki, Finland; 6 Unit of Clinical Physiology, HUS Medical Diagnostic Center, University of Helsinki and Helsinki University Hospital, Helsinki, Finland; 7 Pulmonology, Heart and Lung Center, University of Helsinki and Helsinki University Hospital, Helsinki, Finland; 8 Division of Physiotherapy, Department of Internal Medicine and Rehabilitation, Helsinki University Hospital, Helsinki, Finland; 9 Intensive Care Unit, Meilahti Tower Hospital, Building 1, Haartmaninkatu 4, 00290 Helsinki, Finland

**Keywords:** COVID-19, ARDS, Critical care, Lung function, Exercise capacity

## Abstract

**Background:**

The significant morbidity caused by COVID-19 necessitates further understanding of long-term recovery. Our aim was to evaluate long-term lung function, exercise capacity, and radiological findings in patients after critical COVID-19.

**Methods:**

Patients who received treatment in ICU for COVID-19 between March 2020 and January 2021 underwent pulmonary function tests, a 6MWD and CXR 6 months after hospital discharge.

**Results:**

A restrictive ventilatory defect was found in 35% (23/65) and an impaired diffusing capacity in 52% (32/62) at 6 months. The 6-minute walk distance was reduced in 33% (18/55), and 7% (4/55) of the patients had reduced exercise capacity. Chest X-ray was abnormal in 78% (52/67) at 6 months after hospital discharge.

**Conclusion:**

A significant number of patients had persisting lung function impairment and radiological abnormalities at 6 months after critical COVID-19. Reduced exercise capacity was rare.

**Supplementary Information:**

The online version contains supplementary material available at 10.1186/s12890-022-02023-w.

## Highlights

- A significant number of patients had persisting lung function impairment and radiological abnormalities at 6 months after critical COVID-19.
- Exercise capacity was within normal limits in most patients after critical COVID-19.

## Introduction

Many Coronavirus disease 2019 (COVID-19) survivors suffer from a variety of symptoms after recovery from acute infection [1–6]. The first follow-up studies of COVID-19 survivors have shown persisting symptoms, radiological abnormalities, impaired lung function, and reduced exercise capacity up to 12 months after initial infection [1, 2, 6–10]. Restrictive ventilation and an impaired diffusing capacity have been the most common abnormalities in lung function tests.

As the COVID-19 pandemic continues to cause significant morbidity, it is important to better understand the long-term impacts. It is estimated that 5% of all patients with COVID-19, and 20% of those hospitalised need intensive care support [11, 12]. COVID-19 results in long stays in intensive care unit (ICU) and long periods of immobilisation in prone position [13, 14]. Prolonged invasive mechanical ventilation (IMV) can increase the risk of ventilator-associated events (VAE), such as secondary pneumonia, fluid overload, acute respiratory distress syndrome (ARDS), and atelectasis, which lengthen the hospital stay and increase hospital mortality [15, 16]. Intensive care and IMV can lead to post-intensive care syndrome (PICS) with various cognitive, psychiatric, and physical impairments [13]. Long duration of intensive care, sedation, use of neuromuscular blocking agents and mechanical ventilation, advanced age, and comorbidities, all of which are common features of hospitalised COVID-19 patients, increase the risk of PICS [13].

Acute respiratory failure, as seen in COVID-19, is associated with high mortality [12, 17], and survivors of ARDS are known to suffer from exercise limitation and decreased physical quality of life even 5 years after hospital discharge [18].

Our aim was to evaluate the long-term lung function, exercise capacity, and radiological findings in patients treated in ICU for COVID-19.

## Materials and methods

### Study population

**Fig. 1:**
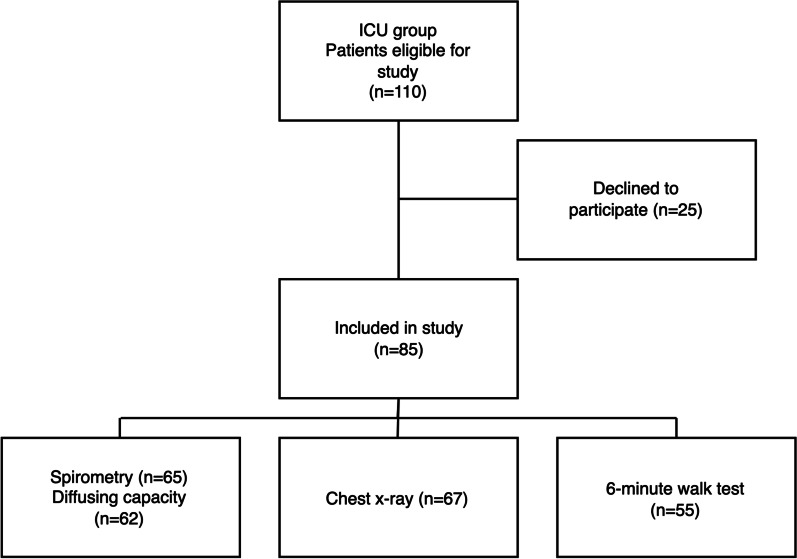
Flow chart of patient selection

All consecutive eligible patients with a laboratory-confirmed (positive SARS-CoV-2- polymerase chain reaction test) COVID-19 treated in ICUs at the Helsinki University Hospital between March 2020 and January 2021 were recruited (Fig. 1). They were identified using the international classification of diseases (ICD-10) code U07.1 (laboratory-confirmed SARS-CoV-2 infection). Two patients from a provincial central hospital also participated in the study. Inclusion criteria for this study were age ≥ 18 years and Finnish as their primary language. We excluded patients with major prior neurological diseases such as Parkinson’s disease, dementia, traumatic brain injury, stroke, and developmental disability due to a concurrent major sub study investigating neuropsychological problems after critical COVID-19. Pregnant patients were excluded. All participants gave their written informed consent for participation in the study, including using data from clinical investigations from their hospitalisation and clinical follow-up.

### Study variables

The primary aims of the study were to assess (1) vital capacity (VC) and forced vital capacity (FVC), (2) percentage predicted diffusing capacity (DLCOc), and (3) 6 minute walk distance (6MWD), all measured 6 months after ICU discharge.

Our secondary aim was to assess the association of eventual impairment in diffusing capacity with patient and treatment-related factors.

### Data collection

Clinical data collected from the hospital admission for SARS-CoV-2 infection included demographic information, medical history, and hospital treatment records as extracted from electronic medical records (Epic™, Verona, US, Uranus™, CGI, Montreal, Canada, PICIS™, Wakefield, US).

Patients were invited to a clinical and research follow-up visit where they were reviewed by a trained intensivist (SK, HO, or JH) 6 months after hospital discharge and underwent pulmonary function tests (spirometry and diffusing capacity), 6MWT, and a chest X-ray (Fig. 1).

#### Spirometry

Spirometry was measured in line with the ATS/ERS guidelines (ATS/ERS 2005) using a Medikro spirometer with software 4.8.0 (Medikro Co, Kuopio, Finland) and interpreted according to Kainu reference values [19]. After the baseline measurements were recorded, patients inhaled 400 μg salbutamol via a spacer (Volumatic^R^, GSK, England) and performed postbronchodilator measurements 15 min later. Spirometry variables evaluated were forced vital capacity (FVC), forced expiratory volume in one second (FEV1), forced expiratory ratio (FEV1/FVC), maximal mid-expiratory flow (MMEF), and maximal flow at 50% of FVC (MEF50).

#### Diffusing capacity

Diffusing capacity was measured using a single-breath method (Jaeger Masterscreen-PFT using Sentry Suite software version 3.1, Vyaire Medical, Würzburg, Germany) using the Viljanen reference values [20] according to recent guidelines [21]. The variables evaluated were total lung capacity (TLC), functional residual capacity (FRC), residual volume (RV), haemoglobin-corrected diffusing capacity for carbon monoxide (DLCOc), and specific diffusing capacity (DLCOc/VA). The mean of two partial measurements was the result of the measurement.

#### 6MWT

6MWT was performed in accordance with the ATS guidelines [22] without supplemental oxygen. The sex-specific equations for predicting 6-minute walk distance (6MWD) were computed using reference equations by Enright and Sherrill [23]. Differences to the walk distance predicted values and lower limit of normal values were calculated for all patients. The lower limit of the normal range (LLN) was obtained by subtracting 153 m from the 6MWD valued for men and 139 m for women. Reduced exercise capacity was defined as 6MWD below 80% of the predicted value. Dyspnoea and fatigue were assessed using the Borg scale (0–10) immediately before and after the test. Heart rate and oxygen saturation (Vyntys™ WALK, Vyaire Medical Inc, Chicago, USA) were recorded online during the 6-min walk and 5 min after stopping.

#### Chest X-ray

Chest X-rays were qualitatively reviewed by an experienced thoracic radiologist (KV) with over 18.5 years’ experience with a specific note of (a) the type of pulmonary abnormality (ground-glass opacities, parenchymal bands or consolidation), (b) laterality of abnormalities (left, right or bilateral) and (c) severity of parenchymal abnormalities (very little, little, some, moderate).

### Ethics

This observational study is a part of the Recovery after critical coronavirus infection (RECOVID) study. The study protocol was approved by the Helsinki University Hospital ethics board (HUS-1949-2020, §148/HUS/1922/2020). This study was conducted in accordance with Good Clinical Practice and the Declaration of Helsinki.

### Statistics

Categorical variables were reported as frequencies and percentages. Continuous variables were reported using median and interquartile range (IQR) for non-parametric data. Comparisons of proportions of categorical parameters between groups were performed using the Chi-square test or Fisher’s exact test when appropriate, the Wilcoxon signed-rank test, and McNemar Test for repeated-measure data. Comparisons of values of non-parametric variables were performed using the Mann–Whitney *U* test for between-group differences. A p-value less than 0.05 was considered statistically significant. For multivariable logistic regression analysis, a forward entry method was chosen using Akaike information criteria (AIC) and Bayesian information criteria (BIC) due to the limited amount of data. Variables with the smallest value of AIC or AIC and BIC together were chosen to be in the final model.

Multivariable analysis was performed using Stata (StataCorp). All other statistical analyses were performed using SPSS version 25 and 27 (IBM SPSS Statistics for Windows and Macintosh, IBM Corp., Armonk, N.Y., USA).

## Results

### Study population

The study population comprised 85 patients, 83 had been treated in Helsinki University Hospital and two in Päijät-Häme Central Hospital. The baseline characteristics of the patients are presented in Table 1 and the clinical characteristics and treatment are in Table 2.

**Table 1:** Baseline characteristics of the 85 patients treated in intensive care units due to a SARS-CoV-2 infection

	N = 85
*Sex*	
Male	52 (61%)
Female	33 (39%)
Age, years	60 (50–68)
BMI, kg/m^2^	30.1 (27.2–34.3)
*Smoking*	
Never	56 (65%)
Former	29 (34%)
Current	0 (0%)
*Comorbidities*	
One or more comorbidities	67 (78%)
Hypertension	49 (58%)
Dyslipidaemia	29 (34%)
Type 1 diabetes	2 (2%)
Type 2 diabetes	18 (21%)
CAD or PAD	12 (14%)
Atrial fibrillation	4 (5%)
Previous DVT/PE or thrombophilia	8 (9%)
Chronic kidney failure	3 (4%)
Asthma	13 (15%)
COPD	1 (1%)
Obstructive sleep apnoea	14 (17%)
Hypothyroidism	5 (6%)
Rheumatoid arthritis	6 (7%)
Gout	5 (6%)
Cancer	2 (2%)
Neurological disease	2 (2%)

Data are n (%) or median (IQR, interquartile range)

BMI, body mass index; CAD, coronary artery disease; PAD, peripheral artery disease; DVT, deep venous thrombosis; PE, pulmonary embolism; COPD, chronic obstructive pulmonary disease

**Table 2:** Clinical characteristics and treatment of the 85 patients treated in intensive care units due to a SARS-CoV-2 infection

	N = 85
SOFA	6 (3–8)^a^
SAPS II	27 (20–38)^b^
APACHE II	17 (13–20)^c^
Max FiO2, % (if not intubated)	60 (40–60)
High flow oxygen delivery	20 (24%)
NIV	3 (4%)
Invasive mechanical ventilation	55 (65%)
ECMO	0 (0%)
Renal replacement therapy	5 (6%)
Prone position	27 (32%)
*Drug treatment*	
Antibiotics	85 (100%)
Oseltamivir^α^	20 (24%)
Remdesivir	2 (2%)
Hydroxychloroquine	0 (0%)
*Corticosteroids*	
Methylprednisolone with ARDS indication^β^	8 (9%)
Dexamethasone	17 (20%)
Other corticosteroids	0 (0%)
*Enoxaparin*	
Prophylactic dose^χ^	68 (80%)
Therapeutic dose^δ^	17 (20%)
Time from symptom onset to hospital admission, days	8 (6–10)
Time from symptom onset to ICU admission, days	10 (8–13)
*Length of ICU stay, days*	
Intubated n = 55	15 (9–25)
Not intubated n = 30	4 (2–6)
All N = 85	9 (5–18)
Duration of invasive mechanical ventilation, days	12 (7–16)
Length of hospital stay, days	20 (15–27)

Data are median (IQR, interquartile range) or n (%)

SOFA, Sequential Organ Failure Assessment Score; SAPS II, Simplified Acute Physiology Score; APACHE II, Acute Physiology and Chronic Health Evaluation; ICU, intensive care unit; FiO2, fraction of inspired oxygen; NIV, non-invasive ventilation; ECMO, extracorporeal membrane oxygenation; ARDS, acute respiratory distress syndrome

^α^Oseltamivir was used during the early pandemic until influenza was excluded and SARS-CoV2-PCR test positivity was confirmed

^β^Methylprednisolone loading dose 1 mg/kg, followed by a continuous infusion of 1 mg/kg/day

^χ^Enoxaparin at prophylactic dose < 1.5 mg/kg/day

^δ^Enoxaparin at therapeutic dose > 1.5 mg/kg/day

Data available for, % ^a^79.6, ^b^79.6, ^c^75.3

### Lung function

Sixty-five patients underwent lung function tests 6 months after hospital discharge (median 180 days (IQR 175–194) (Table 3). A restrictive ventilatory defect was found in 35% (23/65) of the patients. Four patients (6%) had an obstructive ventilatory defect (FEV1/FVC < 0.7), two of whom had a previous asthma diagnosis. One patient had a diagnostic bronchodilator response (FEV1 ≥ 12% and ≥ 200 ml) and did not have a previous asthma/COPD diagnosis. Decreased DLCOc was found in 32 patients (52%), and decreased DLCOc/VA in twelve (19%).

**Table 3:** Lung function and laboratory findings 6 months after initial SARS-CoV-2 infection in patients treated in intensive care units

	6 months	
	Median (IQR)	Decreased value, N (%)
*Spirometry*	n = 65	
FVC Z-score	− 1.1 (− 1.9 to − 0.6)	23 (35)^a^
FEV1 Z-score	− 0.8 (− 1.6 to − 0.2)	15 (23)^b^
FEV1/FVC Z-score	0.9 (− 0.0 to 1.5)	4 (6)^c^
Diagnostic bronchodilator response*		1 (2)
MEF50/MMEF bronchodilator response**		12 (18)
*Diffusing capacity*	n = 62	
FRC % predicted	65 (58–74)	52 (80)^d^
RV % predicted	68 (58–79)	48 (74)^e^
TLC % predicted	85 (76–92)	22 (34)^f^
DLCOc % predicted	79 (70–89)	32 (49)^g^
DLCOc/VA % predicted	93 (83–105)	12 (18)^h^
*Laboratory test results*		
Creatinine	70 (60–81)^i^	
C-reactive protein	< 4 (< 4– < 4)^j,α^	

All values are expressed as median (IQR) or N (%) unless otherwise stated. Spirometry values presented are prebronchodilator measurements

IQR, interquartile range; FVC, forced vital capacity; FEV1, forced expiratory volume in 1 s; FEV1/FVC, forced expiratory ratio; MEF50, maximal flow at 50% of FVC; MMEF, maximal mid-expiratory flow; FRC, functional residual capacity; RV, residual volume; TLC, total lung capacity; DLCOc, diffusing capacity for carbon monoxide corrected for haemoglobin; DLCOc/VA, DLCOc adjusted for alveolar volume

^a^Decreased FVC (Z <  − 1.65)

^b^Decreased FEV1 (Z <  − 1.65)

^c^FEV1/FVC < 0.7

^d^Decreased FRC (< 80% pred)

^e^Decreased RV (< 80% pred)

^f^Decreased TLC (< 80% pred)

^g^Decreased DLCOc (< 80% pred)

^h^Decreased DLCOc/VA (< 80% pred)

^α^Value < 4 is defined as immeasurable low

*Increase of ≥ 12% and ≥ 200 mL in FEV1 or FVC

**Increase of ≥ 36% and 0.5 L/s in MEF50 or ≥ 33% and 0.4 L/s in MMEF

Data available for, ^i^70, ^j^69 cases

### Chest X-rays

**Fig. 2:**
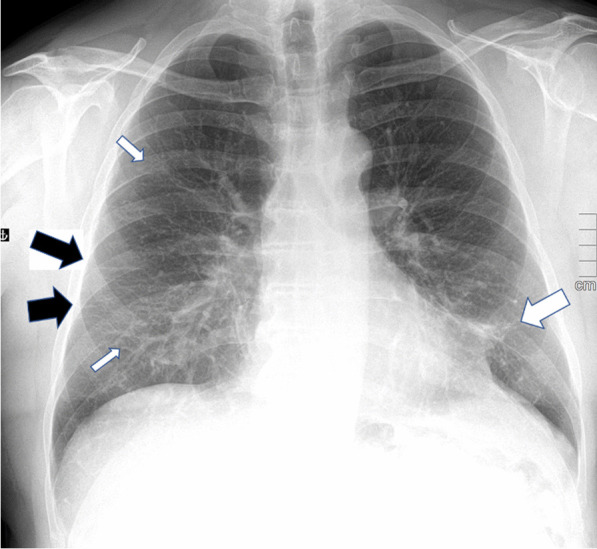
A chest X-ray of an ICU treated patient for COVID-19 showing mainly parenchymal bands (black arrows), faint ground glass opacity (thin white arrows) and consolidation (thick white arrow) 6 months after hospital discharge

Chest X-ray findings are shown in Table 4 and Fig. 2 and Additional file 1: Fig. S1. The median time from hospital discharge to follow-up X-ray was 174 days (IQR 163–181). An abnormal CXR was found in 52/67 (78%) patients at 6 months.

**Table 4:** Chest X-ray at 6 months after hospital discharge

	6 months
N	67
Abnormalities, N (%)	52 (78)
*Quantity of abnormalities, N (%)*	
Very little	30 (45)
Little	18 (27)
Some	4 (6)
Moderate	0 (0)
*Side, N (%)*	
Bilateral	43 (83)
Right	5 (9)
Left	4 (8)
*Type of abnormality*	
Parenchymal bands, N (%)	47 (70)
Ground glass opacities, N (%)	30 (45)
Consolidation, N (%)	3 (5)

### 6-minute walk test

**Fig. 3:**
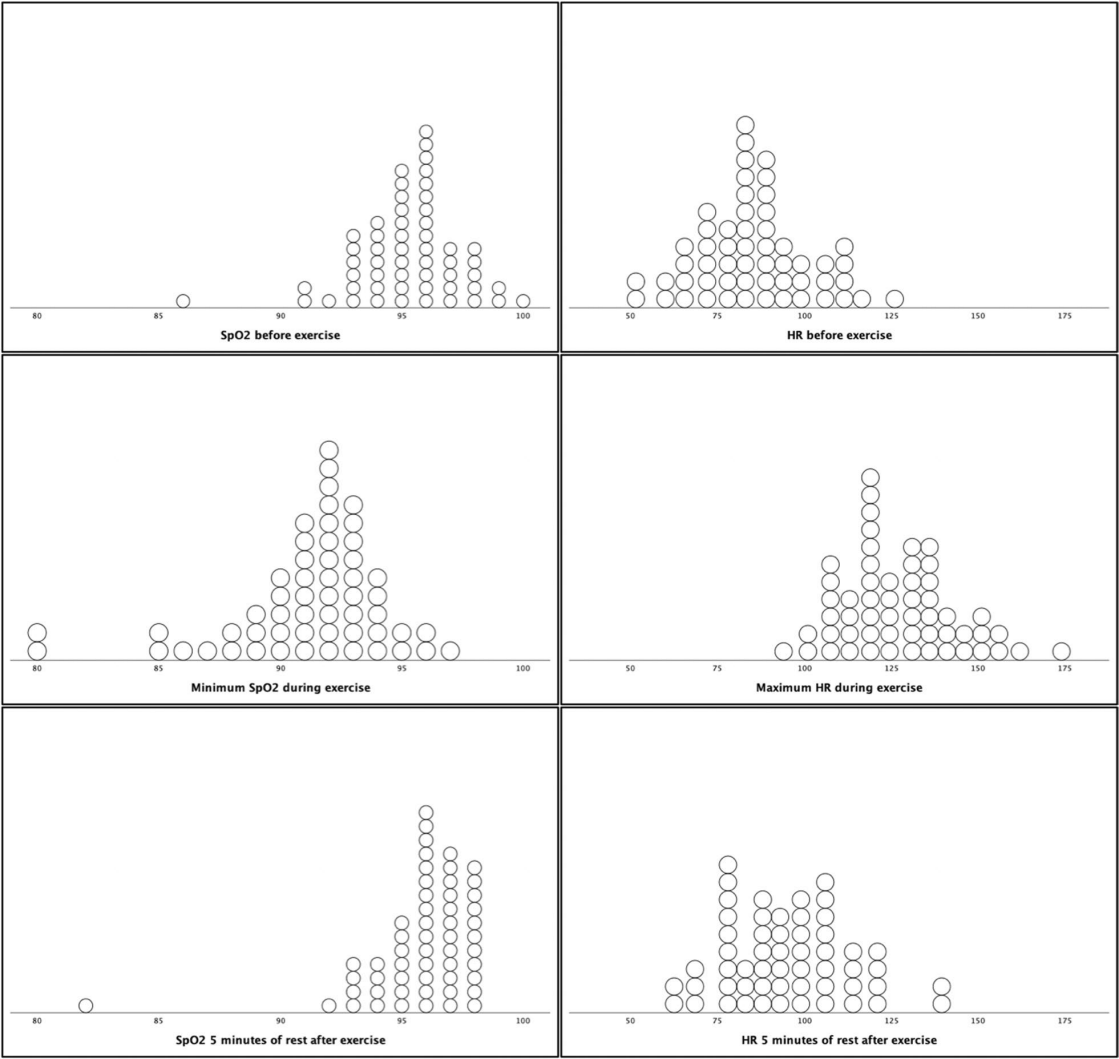
Measured SpO2 and heart rate during the 6-minute walk test. SpO2, peripheral capillary oxygen saturation; HR, heart rate

Fifty-five patients underwent the 6MWT at 6 months after hospital discharge (median 178 days (IQR 171–184) (Table 5, Fig. 3).

**Table 5:** 6-minute walk test 6 months after initial SARS-CoV-2 infection in 55 patients treated in intensive care units

	Value	Range
Walk distance, median (IQR), m	548 (505–607)	135–786
Percentage of predicted value, %, median (IQR)	107.7 (96.3–116.7)	30–142.2
Distance < Predicted value, N (%)	18 (33)	
Distance < 80% of predicted, N (%)	4 (7)	
Distance < LLN, N (%)	2 (4)	
SpO2 before exercise, %, median (IQR)	95 (94–96)	86–100
Minimum SpO2 during exercise, %, median (IQR)	92 (90–93)	80–97
SpO2 5 min of rest after exercise, %, median (IQR)	96 (95–97)	82–98
Dyspnoea on Borg scale before exercise, median (IQR)	0.0 (0.0–1.0)	0–8
Dyspnoea on Borg scale after exercise, median (IQR)	2.0 (0.4–3.0)	0–9
Fatigue on Borg scale before exercise, median (IQR)	0.5 (0.0–2.0)	0–6
Fatigue on Borg scale after exercise, median (IQR)	2.0 (1.4–3.3)	0–8

IQR, interquartile range; LLN, lower limit of the normal range; SpO2, peripheral capillary oxygen saturation; HR, heart rate

**Fig. 4:**
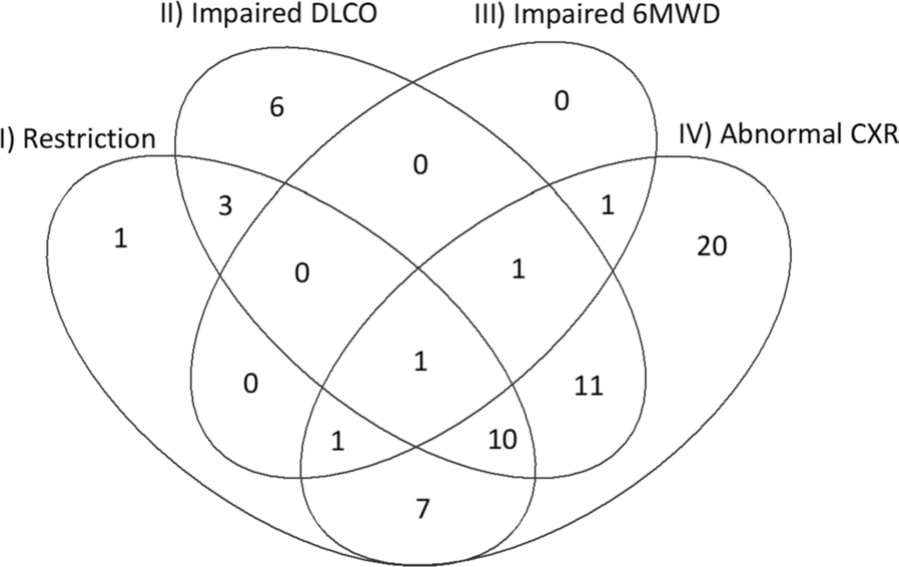
Distribution of abnormal findings in lung function tests, 6-minute walk test and chest X-ray 6 months after hospital discharge

The distribution of abnormal findings in performed tests is illustrated in Fig. 4. We found no association between impaired diffusing capacity at 6 months and reduced exercise capacity in 6MWT (*p* = 0.194), abnormalities in chest X-ray at 6 months (*p* = 0.099), or between reduced exercise capacity and abnormalities in chest X-ray at 6 months (*p* = 0.311) in univariable analysis. In multivariable analysis, impaired diffusing capacity was weakly associated with male sex. Between impaired diffusing capacity and any other included variable (age, BMI, smoking, cardiovascular disease, diabetes, previous DVT/PE, chronic kidney failure, asthma, COPD, obstructive sleep apnoea, intubation, prone position, enoxaparin dose, corticosteroid dose, C-reactive protein at 6 months, or creatinine at 6 months), we found no statistically significant association. Therefore, according to the model building criteria, only male sex was included in the multivariate model. Restriction, defined by FVC z-value under − 1.65, was linked to treatment in prone position, asthma as comorbidity, and male sex.

Numbers represent the number of patients in each group. (I) restriction in spirometry (FVC z-score <  − 1.65); (II) impaired diffusing capacity (DLCOc < 80% of predicted); (III) decreased 6-min walking distance (< 80% of predicted); (IV) abnormalities in chest X-ray.

## Discussion

To our knowledge, this is the first study describing the lung function, exercise capacity, and CXR results in long-term follow-up of Finnish ICU patients. The main findings in our study are that half of the patients had abnormal pulmonary function tests and three fourths of them had abnormalities in CXR at 6 months after hospital discharge. However, the 6MWD was within normal limits in most patients.

One-third of patients with critical COVID-19 had impairment of ventilatory function, and half of these patients (52%) had impaired DLCOc. Restrictive lung function abnormalities and impaired diffusing capacity found in our study are in accordance with other studies, with persistence even up to 1 year post discharge [1, 2, 6, 8, 9, 24–29]. Volume-adjusted diffusing capacity was less often impaired, suggesting diffusing capacity impairment due to restricted lung volume, rather than the damage of lung parenchyma. Previous studies have shown the association between the severity of COVID-19 and restrictive ventilatory defect at follow-up up to 1 year [6, 8]. In addition, impaired diffusing capacity was a common finding among survivors of the ARDS at 6 months follow-up [30, 31].

Persistent radiological abnormalities were common in 78% of our patients at 6 month follow up, with the most common abnormality being parenchymal bands. In previous studies, abnormalities on CXR have been reported up to 7 months after COVID-19 diagnosis [32, 33]. However, in most COVID-19 studies, patients have undergone more in-depth imaging than CXRs.

Despite the impaired diffusing capacity and restrictive pattern in pulmonary function, the exercise capacity was within normal limits in most patients in our study population. The 6-min walk is a commonly used measure of exercise capacity for patients with chronic lung disease, which evaluates the responses of the pulmonary and cardiovascular systems, systemic and peripheral circulation, neuromuscular units, and muscle metabolism [22, 34]. In our study, the distance patients walked in 6 min was longer than expected compared to the previous studies [5, 6, 8, 10, 28, 29]. The walk distance was below the predicted value only in one-third of the patients, only four patients performed reduced exercise capacity assessed as distance below 80% of predicted, and only two patients performed below the LLN. Previous studies have reported that more severe COVID-19 shortened the walk distance up to 120 m at 4–6 months follow-up [1, 6, 25, 29], which is contrary to our finding and makes our results novel. Six-minute walking distance correlates better with physical activity and peak work capacity than the severity of respiratory disease [34]. These data may explain our finding of relatively normal walking distances despite the high prevalence of abnormal pulmonary function tests and residual abnormalities in CXR.

The long-term results in 6-minute walk distance have been studied more thoroughly in association with other severe respiratory conditions: for example, in ARDS survivors the 6-minute walk distance was impaired at 6 months and 1-year follow-up [18, 30, 31, 35]. Our results suggest that after critical COVID-19 the long-term effects on exercise capacity may be less severe compared to other causes of ARDS and other causes of critical respiratory infections [18, 36–38]. This could reflect differences in age, number of comorbidities and organ failures between patients suffering from ARDS related to COVID-19 compared to ARDS of other causes [39, 40]. Severe ARDS can commonly cause prolonged functional disability even 5 years after critical illness [18], but the long-term outcomes after critical COVID-19 are still uncertain.

We found no statistically significant association in multivariable analysis between impaired diffusing capacity at 6 months and any tested variable except male sex, although weakly. In our multivariate model, restriction in spirometry was associated with treatment in prone position, asthma as a comorbidity, and male sex. Male sex and asthma have been previously linked to more severe COVID-19 [1, 41]. This could explain our finding, as disease severity and longer length of stay in the ICU have been associated with poorer long-term lung function in previously published studies [1, 8, 10]. To our knowledge, prone position specifically has not previously been linked to restriction in spirometry in COVID-19 patients. However, treatment in prone position could be considered a marker for disease severity, as prone position was used in patients with the most severe cases of ARDS.

### Study strengths and limitations

The strengths of the study included a detailed examination of a relatively large population of ICU-treated patients. The multi-faceted approach to evaluating these patients using physiological, radiological, and functional outcomes is a major strength to our study.

This study has some limitations. First, our sample size was small. Second, we did not perform an a priori power calculation. Third, participating in the study required one or several visits to the hospital and thus we cannot exclude a selection bias. Fourth, the study population was predominantly overweight, with a median BMI of 30.1. Obesity is associated with smaller lung volumes and a lower DLCOc, while DLCOc/VA is less affected by weight [42]. This could imply that restriction and impaired diffusing capacity in our study population could partially be explained by obesity. However, BMI was not independently associated with decreased DLCOc at 6 months in our analysis. Contrary to the effects on the lung function tests, higher BMI affects the reference value of 6MWT beneficially by reducing it [23]. Considering this, our study patients could have achieved better results in 6MWT due to obesity. However, obesity is also associated with the shorter distance walked in 6 min [43].

Fifth, due to the observational nature of the study, the patients’ lung function before COVID-19 is not known. Thus, we cannot be certain whether the restriction and impaired diffusing capacity we have described are due to COVID-19 or pre-existing lung function impairment. However, only one patient in our sample had previously been diagnosed with COPD, and while 14 patients (15% of the study population) had asthma, asthma rarely causes a restrictive ventilatory defect [44]. Additionally, the patients in our study did not undergo chest CTs, thus allowing us to analyze the radiological abnormalities only to a limited degree. We found that infiltrates on chest x-rays decreased in follow-up from 4 to 6 months, indicating that the findings were reversible albeit slow in progress, thus likely not a result of a pre-existing undiagnosed respiratory condition.

## Conclusions

In conclusion, persisting lung function and radiological abnormalities are common 6 months after severe COVID-19, but the impact on this on exercise capacity is minimal, which is reassuring. Further studies are required to corroborate these findings and assess the long-term trajectory of the abnormalities detected.

## Supplementary Information

**Additional file 1: Figure S1**. Grading of pulmonary abnormalities in patients with history of COVID-19 pneumonitis.

## Data Availability

Data are not publicly available, because consent for public data sharing was not included in the original consent formulation. Data can be partially shared (without data enabling identification) within the EU/EESC upon request for research purposes. For data sharing, contact Salla Kattainen.

## Abbreviations

ARDS: Acute respiratory distress syndrome
ATS/ERS: American Thoracic Society/European Respiratory Society
BMI: Body mass index
CAD: Coronary artery disease
COPD: Chronic obstructive pulmonary disease
COVID-19: Coronavirus disease 2019
DLCOc: Haemoglobin-corrected single breath diffusing capacity for carbon monoxide
DLCOc/VA: Specific diffusing capacity (volume-adjusted DLCOc)
DVT: Deep venous thrombosis
ECMO: Extracorporeal membrane oxygenation
FEV1: Forced expiratory volume in one second
FEV1/FVC: Forced expiratory ratio
FiO2: Fraction of inspired oxygen
FRC: Functional residual capacity
FVC: Forced vital capacity
ICU: Intensive care unit
IMV: Invasive mechanical ventilation
LLN: The lower limit of the normal range
MEF50: Maximal flow at 50% of FVC
MMEF: Maximal mid-expiratory flow
NIV: Non-invasive ventilation
PAD: Peripheral artery disease
PE: Pulmonary embolism
PICS: Post intensive care syndrome
RV: Residual volume
SARS-CoV-2: Severe acute respiratory syndrome coronavirus
TLC: Total lung capacity
VAE: Ventilator-associated event
VC: Vital capacity
6MWT: 6-minute walk test
6MWD: 6-minute walk distance
