# Supplementary material for: Lung function and exercise capacity 6 months after hospital discharge for critical COVID-19
Source: BMC Pulm Med. 2022 Jun 22;22:243. doi: 10.1186/s12890-022-02023-w (PMC9215155; doi:10.1186/s12890-022-02023-w)
Supplement: Supplementary file 1 — Additional file 1: Figure S1. Grading of pulmonary abnormalities in patients with history of COVID-19 pneumonitis. [file 12890_2022_2023_MOESM1_ESM.pdf]

Supplementary figure 1. Grading of pulmonary abnormalities in patients with history of COVID-19 pneumonitis

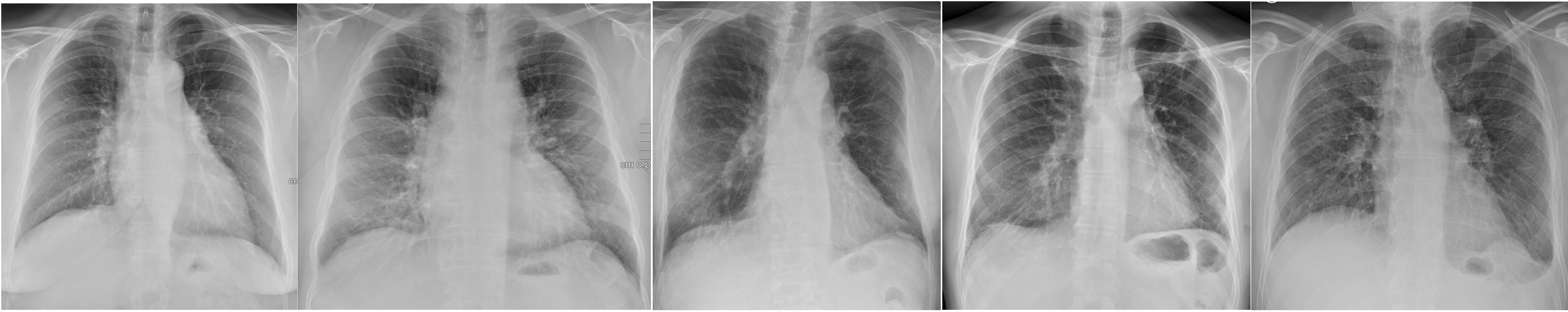

0 = Normal with no abnormalities

1 = Very little

2 = Little

3 = Some

4 = Moderate
